# Non-Cystic Fibrosis Bronchiectasis in Pediatric Age: A Case Series in a Metropolitan Area of Northern Italy

**DOI:** 10.3390/children9091420

**Published:** 2022-09-19

**Authors:** Marcella Gallucci, Egidio Candela, Emanuela Di Palmo, Angela Miniaci, Andrea Pession

**Affiliations:** 1 Pediatric Unit, IRCCS Azienda Ospedaliero-Universitaria di Bologna, 40138 Bologna, Italy; 2 Specialty School of Pediatrics, Alma Mater Studiorum, University of Bologna, 40126 Bologna, Italy

**Keywords:** non-cystic fibrosis bronchiectasis, lung exacerbations, airway clearance techniques, recurrent lower airway infections, protracted bacterial bronchitis

## Abstract

Non-cystic fibrosis bronchiectasis is an emergent disease characterized by endobronchial suppuration, dilated airways with neutrophilic inflammation and chronic wet cough due to recurrent lower airway infections. A regular clinical follow-up and adequate management of exacerbations are essential to reduce symptoms and the worsening of lung injury. We report a retrospective study comprising 15 children and adolescents with NCFB followed in our hospital center of pediatric pulmonology. We retrospectively analyzed the main comorbidities associated with the presence of NCFB, the radiological aspect associated with the different etiologies and the therapeutic approach used. We also emphasized the importance of an effective preventive strategy to reduce and prevent pulmonary exacerbations.

## 1. Introduction

Bronchiectasis is the result of a chronic pulmonary disease characterized by an abnormal, progressive, and often irreversible process of bronchial dilation. These structural changes of the bronchial wall are mainly due to recurrent infectious that lead to chronic airway inflammation. Cystic fibrosis (CF) is the most common cause of bronchiectasis in children while non-cystic fibrosis bronchiectasis (NCFB) is associated with a widespread variety of conditions [1]. The real incidence of NCFB is probably underestimated worldwide, recent data suggests a prevalence ranges from 0.2 to 735 cases per 100,000 children with increased prevalence among lowest socioeconomic classes in developing countries [2]. This connection seems to be directly related to the poor living conditions that predispose to more frequent infectious episodes. In industrialized countries the diagnosis of NCFB is progressively more frequent probably due to the advances in the diagnostic technique [3]. There are several conditions that can cause NCFB and the underlying causes in children are still not completely clear. The most common risk factor of pediatric bronchiectasis is a history of recurrent lower airway infections. Others causes include a dysregulation of immune function (primary or acquired), gastroesophageal reflux (GER) with chronic aspiration, primary ciliary dyskinesia (PCD), foreign body aspiration and congenital anomalies [4,5,6,7,8].

The clinical presentation of NCFB can be variable. The most common symptom during first evaluation is a chronic wet cough. Other symptoms include dyspnea, recurrent wheezing, persistent purulent expectoration, hemoptysis, growth failure and chest wall deformities [9]. The definitive diagnosis of bronchiectasis requires the chest high-resolution computerized tomography (HRCT) while chest radiography has a limited diagnostic value [10]. In 2021, a specific Task Force of the European Respiratory Society developed guidelines for the management of pediatric bronchiectasis providing practical evidence-based management strategies [11]. A correct diagnostic path is the first step to identify the potentially treatable causes, including specific immune deficiency treatment, appropriate therapy for gastro- esophageal reflux or surgery for congenital malformations. Overall airway clearance techniques (ACTs) and antibiotics are considered the milestones of NCFB treatment [12,13]. There is an increased interest about the use of macrolides that seems to allow a significant reduction in frequency of exacerbation [14,15].

We report a case series comprising 15 children and adolescents with NCFB, emphasizing the etiology, clinical characteristics and therapeutic approach used in these patients.

## 2. Materials and Methods

We retrospectively collected 15 patients with a diagnosis of NCFB referring to our pediatric center of Scientific Institute for Research and Healthcare Sant’Orsola University Hospital in Bologna (Italy), from March 2009 to December 2020. The main inclusion criteria for selection were diagnosis of NCFB confirmed by chest HRCT, the age of less than 18 years at the time of diagnosis, availability of demographic and clinical data for each patient. Exclusion criteria included: age over 18 years at diagnosis, the concomitant diagnosis of Cystic Fibrosis based on a genetic analysis, diagnosis of neuromuscular disease with chronic aspiration. Informed consent was obtained from the patients’ parents. We analyzed demographic characteristics of the studied population, including age, gender, birth weight, gestational age, and birthplace. From the medical records we reviewed: associated comorbidities, laboratory findings, pulmonary function test, clinical features and medical treatments. Among associated conditions, atopy was defined either as a sensitization to one or more allergens measured by SPT or the presence of serum allergen specific IgE antibodies. The collected data refer to the time of the first clinical evaluation when the diagnostic path started, while the spirometric data were collected after the diagnosis during the clinical follow up, according to the age of the patient. All subjects underwent chest HRCT and bronchiectasis grading was scored using a modified Bhalla score [16,17,18] and according to Webb et al., therefore a bronchiectasis was confirmed for a broncho-arterial ratio > 0,8. Follow-up for NCFB in our clinic includes one visit every 3–6 months in the absence of exacerbations. A respiratory exacerbation was defined as a worsening of wet cough and increased sputum purulence and quantity for at least 3 days [11].

The spirometry data were analyzed according with the American Thoracic Society/European Respiratory Society criteria [19]. Patients who experienced an episode of wheezing or dyspnea (shortness of breath) and with spirometric indices suggestive of an airway obstruction, underwent a bronchodilator response test (BRT) with Salbutamol. According to GINA guidelines, the test was considered positive if the variation in the forced expiratory volume in one second (FEV1) after bronchodilator administration is ≥12% [20]. The diagnostic path in our center also includes the exclusion of immunodeficiencies initially through the evaluation of the serum concentration of immunoglobulins, lymphocyte typing and response to vaccinations. Assessment of ciliary ultrastructure was performed by transmission electron microscopy (TEM). All collaborating children (14/15) performed a sputum culture at the first evaluation and during exacerbations. The t-test was used to compare the number of exacerbations before and after the start of antibiotic therapy with Azithromycin. Data on exacerbation rates and lung localization was compared by chi square test. This last was also used to compare different pathogens detected in sputum culture and different lung lesion sites.

## 3. Results

Our population includes 15 patients (9 boys, 6 girls). Only one patient was born preterm. Birth weight for all newborns was over 2000 g. Most of patients (13/15) were born in Italy. The patients’ median age at the diagnosis was 8 years (range: 1–19 years).

The most common cause of NCFB in our cohort was previous infection (53,3% of cases, 8 children), followed by primary immunodeficiency (13.3%, 2 patients), GER (6.6%) PCD (6.6%), heart disease (6.6%) (Table 1). 

The main detected conditions associated with NCFB were atopy in 6 children, esophageal atresia (III type) in 1 case of post-infectious NCFB, lung neuroblastoma in 1 patient with post-infectious NCFB, right-sided aortic arch with compression by the anonymous trunk in 1 girl, ventricular septal defect in another baby with a suspect syndromic disease (Table 1).

The time from the onset of respiratory symptoms and the diagnosis of bronchiectasis widely varied with average time of 50 months. The most frequent symptom referred to the medical history was wet cough (Table 2).

Two of the 15 patients had previously been surgically operated on thoracic area. One at the age of 15 months for a left thoracic neuroblastoma, the other due to a tracheal compression by the anonymous trunk underwent aortopexy of ascending and descending aortic limb. The median duration of follow up was 7,13 years (range 3–18).

Pulmonary function tests were performed in all patients from the age of 5 and subsequently repeated every 3–6 months. Spirometry revealed in 4 cases a mixed disventilatory syndrome, 1 patient had a restrictive pattern and 1 other an obstructive one. The others had normal flow-volume curve. Bronchial hyperresponsiveness, documented by a BRT, was found in 6 cases (3 cases of post infectious NCFB, the girl with PCD and 2 patients with immunodeficiency).

Only in one case bronchial hyperresponsiveness was documented during an acute inflammatory event.

A nasal brushing with ultrastructural analysis of cilia was performed in 14/15 patients while the patient with CVID did not perform this diagnostic test.

A diagnosis of PCD was obtained in one case. Secondary ciliary alterations were detected in 12 patients. Microbial exams on sputum samples showed a prevalence of non-typeable Haemophilus Influenzae colonization (5 children, 33%), followed by Pseudomonas A. in one case and S. Aureus in another. Only 2 patients performed a bronchoscopy. In both children, bronchoalveolar lavage (BAL) culture was positive for Haemophilus Influenzae (at a concentration higher than 10^6^) and in one boy Pseudomonas A. was detected.

The BAL analysis of patient with GER presented cylindrical cells of the bronchial epithelium, granulocytes, lymphocytes, numerous histiocytes, some of which positive for RED-OIL staining, compatible with a cytological picture of reactive inflammation. The boy repeated bronchoscopy two years later, and the finding of numerous macrophage elements positive for RED-OIL staining was confirmed. He also underwent an esophageal Impedance-pH monitoring that showed a reflux index of 54%.

One boy showed in the genetic sequencing for cystic fibrosis the pathogenetic variant c.350 > A in heterozygosis.

Regarding the radiological findings detected by HRCT, the distribution of the bronchiectasis was unilateral in 8 cases, bilateral in 7 (46% of cases).

Cylindric bronchiectasis were detected in nine subjects (60%), the girl with the diagnosis of PCD had varicose bronchiectasis, 1 patient had cystic pattern while the others showed heterogenous aspects (bronchial dilatation with bronchial wall thickening and lack of normal bronchial tapering). The lower left lobe was most frequently affected (7 patients). CT findings are shown in Table 3.

In 8 subjects, chest X-ray performed before diagnosis showed non-specific findings such as parenchymal thickening. Regarding the management and prevention of exacerbations, 13/15 of our patients regularly used a positive expiratory pressure (PEP) mask 2–3 times per day.

After the diagnosis, all children started an antimicrobial prophylaxis with Azithromycin, 3 days per week during cold months in patients with recurrent exacerbations (5 subjects) or 3 days on alternate weeks for patients with few exacerbations (<3 per year).

In 5 children, antibiotic therapy was continued for a period > 24 months, in consideration of the clinical history of frequent exacerbations. The mean duration of Azithromycin antibiotic therapy was 26.7 months. The median number of exacerbations at time of diagnosis was 3.4 for year. A significant reduction of the number of exacerbations was observed after starting Azithromycin treatment (mean of 0.7 exacerbations for year, *p* < 0.000001). One boy regularly used anti-reflux therapy with omeprazole 20 mg twice a day.

Moreover, 13/15 patients received a prophylactic inhaled corticosteroids (ICS) treatment, in five cases in association with a long-acting beta-agonists (LABA). 

The choice to use ICS therapy was linked to the frequent finding of wheezing during exacerbations. Using the Pearson’s Chi-Square test no correlation was found between sputum/BAL culture positive for pathogenic organisms and lesions localization (*p* = 0.14). Bilateral involvement of lung (46%) was observed in seven subjects while eight children had NCFB in lower left lobe. No significant lobar predominance was seen in any of the etiology-groups (*p* < 0.2). We did not observe a statistically significant correlation between the localization of bronchiectasis and the underlying pathology as well as between bilateral/unilateral localization and frequency of exacerbations (*p* < 0.14). The girl with PCD had varicose bronchiectasis involving both lung fields.

This 12-year-old girl was born in Pakistan from consanguineous parents, and she moved to Italy when she was 3. Parents reported healthy conditions up to the age of 10 except for several recurrent episodes of wet cough often treated with antibiotic therapy with transient benefit.

The girl came to our attention at the age of 11. She showed a normal immunologic screening, while the HRCT revealed numerous bronchiectasis with prevalent diffusion on the right lung. Therefore, she underwent a nasal brushing that identified an ultrastructural pathological change compatible with a PCD (116 cilia of which 95 pathological). 

Detected cilia changes were absence of the central pair (64 cilia), absence of a microtubule of the central pair (5 cilia), absence of a pair of peripheral microtubules (6 cilia), assonema disorganization (9 cilia), presence of supernumerary microtubules (3 cilia), swollen cilia (8 cilia). After the diagnosis, the girl started prophylaxis with 3-day weekly Azithromycin and a daily PEP mask treatment with significant improvement of the wet cough. No exacerbation was reported 3 months after the start of therapy.

No patients underwent surgical treatment (such as segmentectomy or lobectomy) for their NCFB.

## 4. Discussion

In our cohort the most common detected cause of bronchiectasis was a history of lower airway infections as 9 children had a diagnosis of “post infectious” bronchiectasis. A previous PBB was present only in 1 child. Sputum culture showed the presence of Hemophilus Influenzae (10^6^). We hypothesized a diagnosis of Protracted Bacterial Bronchitis (PBB), we decided to undertake antibiotic therapy which was continued for 4 weeks with clinical improvement and subsequent sputum culture negativity. Nevertheless, due to the reappearance of wet cough one month after stopping therapy, we decided to perform a chest CT which showed the presence of small bronchiectasis in the lower right parahilar side and in the lower left lobe. As it is known PBB can cause a persistent endobronchial neutrophilic inflammation, known risk factors for the onset of NCFB, so that the PBB and NCFB may represent different stages of a single disorder [21,22].

A failure to improve a wet cough after a 4-weeks antibiotic therapy requires more investigations (such as CT scan, bronchoscopy, immunity tests) to rule out the presence of another underlying disease [23]. In our case, the early diagnosis of NCFB allowed a mild and well-controlled course of the disease since the bronchiectasis detected by HRCT were at a very early radiological stage.

Subsequently the boy started a daily treatment with PEP mask and cycle of 3 days a week of Azithromycin (for 6 months) with improvement of symptoms and no further coughing episodes reported. A clinical follow-up every 2 months was set up to monitor general conditions. Except for the presence of Haemophilus I. in sputum culture, no other relevant risk factors for NCFB emerged. Wurzel et al. in their prospective longitudinal cohort study involving 161 children with PBB identified two significant risk factors for bronchiectasis: recurrent PBB (>3 episodes/years) and the detection of H. influenzae infection in the lower airways [22]. Authors also showed that approximately one of 12 children with PBB had diagnosis with bronchiectasis at 2-years follow-up, with many experiencing recurrent episodes of PBB. This study provides further evidence to support a link between PBB and bronchiectasis and could suggest the need to monitor children with PBB over time and to consider chest CT imaging in those with risk factors for bronchiectasis. About the case of PCD, noteworthy is that the genetic analysis performed by next generation sequencing (NGS), was not decisive for the diagnosis. There is still a partial understanding of all genetic mutations involved in the PCD that certainly limits the sensitivity of genetic testing, therefore it’s not often sufficient on its own for the final diagnosis [24]. It is estimated that genetic examination fails to identify approximately 30% of diagnosis of PCD, also because many individuals may have variants of uncertain significance or mono-allelic heterozygous variants that need confirmation by functional and structural tests [25]. The main ultrastructural finding detected in our case was the presence of defects of central microtubule pair (CP) in 64 cilia.

At present, only two genes, HYDIN (HYDIN axonemal central pair apparatus protein) and STK36 (serine/threonine-protein kinase 36), have been described to cause CP defects in PCD [25]. The central pair (CP) complex projections are often small and hard to detect due to the resolution limits of diagnostic electron microscopes [26]. Moreover, some evidence showed that obtaining a diagnosis of PCD in subjects with central pair (CP) defects is even more difficult, since mutations involving etiology of these defects (such as mutations of HYDIN) are difficult to identify. Indeed, the genetic analysis of HYDIN variants may be confounded by the pseudogene HYDIN2, which have a structure almost identical [27]. In our case the diagnosis of PCD was obtained considering the suggestive clinical history and the other structural cilia alterations too. Furthermore, this girl didn’t have a history of neonatal distress or recurrent otitis/sinusitis, and chronic wet cough was the only symptoms reported by parents. This example demonstrates how the clinical presentation of PCD can be very heterogeneous and variable. Between our cohort of patients, one presented bronchiectasis likely associated with gastroesophageal reflux documented by esophageal impedance-Ph monitoring. In literature there is still no reliable evidence of the association between gastroesophageal reflux and bronchiectasis [28]. 

Nevertheless, gastro-esophageal reflux disease (GERD) is a frequent comorbidity in bronchiectasis, and it may be correlated with poorer outcomes. The prevalence of GERD in bronchiectasis ranges from 26% to 75% [28]. Several factors have been implicated to explain this correlation including evidence of pulmonary micro-aspiration of gastric contents. Moreover, subjects with bronchiectasis and GERD often exhibit increased mortality and bronchiectasis severity, with more recurrent exacerbations, hospitalizations, reduced pulmonary function and poorer quality of life. For these reasons, detecting GERD in patients with NCFB may have a potentially relevant role in the therapeutic approach, although to date other clinical evidence are needed [15,28]. In our case, the association of antireflux therapy with antibiotic prophylaxis with Azithromycin, allowed for adequate control of pulmonary exacerbations. Among our patients five children also had a history of recurrent wheezing during first years of life, many patients (73%) experienced wheezing during exacerbations and 6 had a diagnosis of asthma. Several studies have shown that, in daily medical practice, ICS with or without LABA, are commonly prescribed in patients with NCFB even without a diagnosis of coexisting asthma [29].

Children with bronchiectasis often develop wheeze or asthmatic symptoms, with rates ranging from 11 to 46% although it is not always clear if this finding derives from a concomitant asthma, or it is the result of a bronchial hyperreactivity linked to bronchiectasis (30). In our patients, the choice to use ICS was probably guided by the observation, not always quantifiable, of an advantage in symptom control, although an accurate assessment of clinical characteristics of children who could benefit from ICS should be performed before starting this treatment [30]. Children with bronchiectasis have recurrent acute lung exacerbations and often they require hospital admission when oral therapies fail. Moreover, a clinical history of recurrent exacerbations may lead to progressive deterioration of lung function, so it is one of the strongest predictors of poor quality of life among children with bronchiectasis [31,32]. 

The antibiotic therapy is a key intervention in the management of NCFB. Antimicrobial interventions can be used in two different ways: in the treatment of an acute exacerbation or as prophylaxis to reduce the frequency of acute events. In general, the use and choice of antimicrobials should target the specific pathogens and the treatment duration should be decided in relation with the frequency of exacerbations, disease severity and response to previous therapy. Ideally, a sputum sample culture or nasopharyngeal aspirates (in children unable to expectorate) should be obtained prior to initiating antibiotics [32].

The British Thoracic Society bronchiectasis guidelines suggest that all children and adults with bronchiectasis should have an assessment of lower respiratory tract microbiology [33]. In our series it was possible to perform a sputum culture in only 7 children due to the inability of many children to expectorate. All our patients received an antimicrobial prophylaxis with Azithromycin. The use of antimicrobial prophylaxis may be suitable in situations where frequent exacerbations are expected to occur [34], for this reason we decided to extend the antibiotic prophylaxis in those children with more than 3 exacerbations per year (6 children).

The use of macrolides for the treatment of NCFB has become a common approach in recent years, thanks to several characteristics: their anti-inflammatory effects, their ability of decrease mucus production and the well-known effect on Gram-positive cocci and atypical pathogens. Recent evidence suggested that long-term treatment with macrolides, in particular Azithromycin, significantly reduced the incidence of non-CF bronchiectasis exacerbations improving quality of life, even though many studies concern the adult population [12,35]. Among pediatric patients only one long-term, randomized double-blind placebo-controlled trial was conducted and showed that once-weekly Azithromycin for up to 24 months decreased pulmonary exacerbations among Indigenous children with non-cystic-fibrosis bronchiectasis [36].

Kobbernagel et colleagues in a recent double-blind, randomized, placebo-controlled phase 3 trial including patients with diagnosis of PCD, showed that participants receiving Azithromycin had significantly lower rate of exacerbations during the therapy periods [37]. Among our cases too, after starting Azithromycin prophylaxis there was a significant reduction in the number of lung exacerbations (3.4 per year versus 0.7; *p* < 0.000001) 

Nevertheless, despite several encouraging evidence, the prolonged use of antibiotic prophylaxis can cause an increased risk of the emergence of resistance to antibiotics that should not be underestimated [12,38]. For these reasons, an accurate profile of children who could benefit from this therapy needs to be established and further studies are required to do so. In his recent paper A. Bush too focused on risk of antibacterial resistance “if macrolides are used widely and uncritically in the community” stating that Azithromycin is not the answer to anything in pediatric respiratory medicine [39].

Finally, most of our patients (13/15) habitually used a PEP mask 2–3 times per day with increased frequency of sessions during exacerbations. Among our patients we were unable to quantify the effectiveness of ACTs on the number of exacerbations, as the time of use was very variable among different subjects. Nevertheless, use of Airway clearance techniques (ACTs) is one of the milestones for NCFB management and are recommended in children with NCFB (11). Most of studies about ACTs, including many active cycles of breathing technique, chest physiotherapy, devices that create a positive pressure (PEP mask or high-pressure PEP therapy) or high-frequency chest wall oscillation, mainly investigate patients with cystic fibrosis [40,41]. Currently there is no consensus regarding the superiority of one ACTs technique over another. 

In some studies, the application of positive expiratory devices and high-frequency chest wall oscillation showed an improvement of FEV1, while a recent comparison between the effects of short and medium-term use of PEP therapy to other forms of ACTs showed no greater advantage in sputum expectoration and no difference in lung function tests [41]. Nevertheless, these studies were conducted in patients in a stable clinical state, therefore it’s still uncertain the effects of ACTs during an acute episode and further studies are needed, especially in children. In the pediatric population an appropriate training of the patient and the family on the use of different ACTs is mandatory.

**Figure 1:**
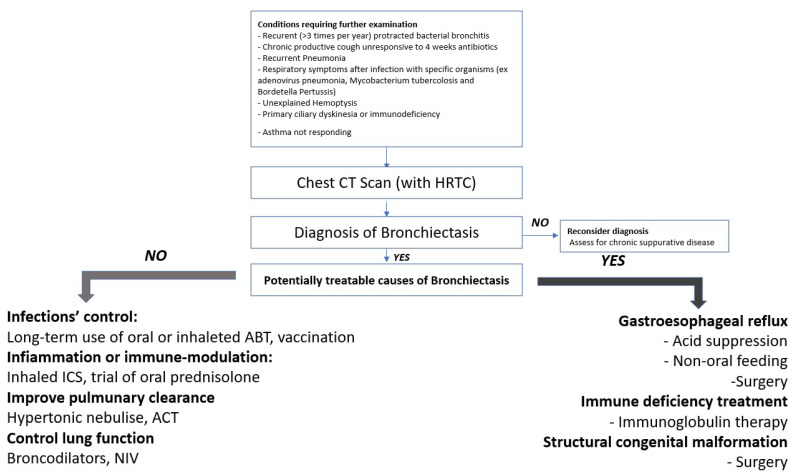
Management of NCFB.

Positive expiratory pressure physiotherapy through a face mask is a simple and effective tool that can also be used by small children [42]. We have inserted a flow-chart in order to propose a management of an NCFB case (Figure 1).

In our cases it has proved very useful to prevent bronchial mucous encumbrance. Moreover, both patients and families maintained good adherence to therapy despite it having been performed for a long time.

## 5. Conclusions

Despite the small number of subjects, we observed that a close follow up, an adequate care and an early treatment (comprising antibiotic prophylaxis with weekly Azithromycin and daily ACTs) can reduce the exacerbation rates and the recurrence of wet cough, which is certainly a disabling symptom for both families and children.

Certainly, a limitation of this study is that it is purely observational. Moreover, due to the small number of patients, some of emerged data did not reach a statistical significance. A greater evidence base is required in the future to improve clinical outcomes and prognosis of children with bronchiectasis.

## Figures and Tables

**Table 1:** Associated conditions. Some patients have more than one associated condition.

Associated Conditions	Number of Patients
Previous lower airways infections	8
Primary ciliary dyskinesia	1
Immune deficiency	2
Gastroesophageal reflux disease	2
Right-sided aortic arch	1
Syndromic unknown disease	1
Lung neuroblastoma	1
Asthma/atopy	6
Protracted Bacterial Bronchitis	1
Respiratory distress or intubation during perinatal period	2

**Table 2:** Clinical patient’s characteristics.

Symptoms	Number of Patients
Recurrent wheezing in first year of life	5
Recurrent wheezing	11
Wet Cough	15
Dyspnea	1
Persistent purulent expectoration	4
Recurrent infections of lower respiratory tract	15
Sinusitis	2
Laryngotracheitis	1
Hemoptysis	0

**Table 3:** Radiologic findings. PI: Post infectious; NB: Neuroblastomas; EA: Esophageal atresia; CP: cardiopathy, ID: Immune-deficiency.

Radiologic Findings	N patients	Associated Conditions (Number Subjects)	Localization
Cystic	1	GER	lower left lobe
Cylindric	9	PI (5) CVID/EVANS (1) NB (1) EA (1) CP (1)	2 medium lobe, 2 lower left lobe, 1 right lower lobe bilateral Lower and apical left lobe lower left lobe medium lobe
Varicose	1	PCD (1)	medium lobe, lower right lobe, lower left lobe
Non-specific morphology	4	ID (1) PI (3)	medium lobe 1 lower left lobe, 1 bilateral upper lobe 1 medium lobe and lower left lobe

## Data Availability

All clinical data and material are available in our Pediatric Unit.
